# Multiple Recombination Events and Strong Purifying Selection at the Origin of SARS-CoV-2 Spike Glycoprotein Increased Correlated Dynamic Movements

**DOI:** 10.3390/ijms22010080

**Published:** 2020-12-23

**Authors:** Massimiliano S. Tagliamonte, Nabil Abid, Stefano Borocci, Elisa Sangiovanni, David A. Ostrov, Sergei L. Kosakovsky Pond, Marco Salemi, Giovanni Chillemi, Carla Mavian

**Affiliations:** 1 Emerging Pathogen Institute, University of Florida, Gainesville, FL 32608, USA; mstagliamonte@ufl.edu; 2 Department of Pathology, Immunology and Laboratory Medicine, University of Florida, Gainesville, FL 32610, USA; ostroda@pathology.ufl.edu; 3 Laboratory of Transmissible Diseases and Biological Active Substances LR99ES27, Faculty of Pharmacy, University of Monastir, Rue Ibn Sina, 5000 Monastir, Tunisia; nabilabidbensalem.2014@yahoo.fr; 4 Department of Biotechnology, High Institute of Biotechnology of Sidi Thabet, University of Manouba, BP-66, 2020 Ariana-Tunis, Tunisia; 5 Department for Innovation in Biological, Agro-food and Forest Systems (DIBAF), University of Tuscia, via S. Camillo de Lellis s.n.c., 01100 Viterbo, Italy; borocci@unitus.it (S.B.); elisa.sangiovanni93@gmail.com (E.S.); 6 Institute for Biological Systems, National Research Council, Via Salaria, Km 29.500, 00015 Monterotondo, Rome, Italy; 7 Department of Biology, Temple University, Philadelphia, PA 19122, USA; tug23285@temple.edu; 8 Institute of Biomembranes, Bioenergetics and Molecular Biotechnologies (IBIOM), National Research Council, Via Giovanni Amendola, 122/O, 70126 Bari, Italy

**Keywords:** SARS-CoV-2, COVID-19, recombination, furin-like cleavage site, ACE2, S glycoprotein, molecular dynamics, bioinformatics

## Abstract

Our evolutionary and structural analyses revealed that the severe acute respiratory syndrome (SARS) coronavirus 2 (SARS-CoV-2) spike gene is a complex mosaic resulting from several recombination events. Additionally, the fixation of variants has mainly been driven by purifying selection, suggesting the presence of conserved structural features. Our dynamic simulations identified two main long-range covariant dynamic movements of the novel glycoprotein, and showed that, as a result of the evolutionary duality, they are preserved. The first movement involves the receptor binding domain with the *N*-terminal domain and the *C*-terminal domain 2 and is maintained across human, bat and pangolin coronaviruses. The second is a complex network of long-range dynamics specific to SARS-CoV-2 involving the novel PRRA and the conserved KR*SF cleavage sites, as well as conserved segments in *C*-terminal domain 3. These movements, essential for host cell binding, are maintained by hinges conserved across human, bat, and pangolin coronaviruses glycoproteins. The hinges, located around Threonine 333 and Proline 527 within the *N*-terminal domain and *C*-terminal domain 2, represent candidate targets for the future development of novel pan-coronavirus inhibitors. In summary, we show that while recombination created a new configuration that increased the covariant dynamic movements of the SARS-CoV-2 glycoprotein, negative selection preserved its inter-domain structure throughout evolution in different hosts and inter-species transmissions.

## 1. Introduction

Coronavirus disease 2019 (COVID-19) is a severe acute respiratory syndrome (SARS), caused by a newly emerged human coronavirus (CoV-2) strain (SARS-CoV-2). CoVs are positive sense single-stranded RNA viruses infecting a broad range of hosts [1], and have shown the ability to shift from a natural reservoir and cause outbreaks in human populations [2]. CoV genomes encode for a methyltransferase with 3’-5’ exonuclease activity; this constitutes a proofreading and mismatch correction system, resulting in lower mutation rates compared to other RNA viruses [3,4,5]. This feature might ultimately limit the mutagenic variability of the virus, and enhance the prominence of recombination in its evolution and expansion of species and cellular tropism [6,7]. Recombination is an important factor driving viral diversity [2,8,9,10,11,12,13], and it has been reported as a possible mechanism favoring cross-species CoVs transmission as well as increasing their adaptability to a new host [2,8]. Early studies that investigated the origin of SARS-CoV-2 based only on similarity plots analyses showed evidence of a recombination event, involving SARS-CoV-2 spike (S) glycoprotein, between a bat isolate RaTG13 (Bat-CoV-RaTG13) and a CoV from pangolin (Pangolin-CoV-2019), as progenitors of SARS-CoV-2 [14,15]. A recent study has also shown a new CoV strain, RmYN02, having amino acid insertions at the S_1_/S_2_ cleavage site similar to the insertions in the SARS-CoV-2 glycoprotein; however, the overall low similarity between strains point to an independent origin of these insertions [16]. These findings are relevant since recognition and binding to the host cell surface receptor is mediated by the S glycoprotein homotrimeric complex [17]. Previous work using similarity plot to detect recombination may have underestimated the number of recombination events that shaped SARS-CoV-2 S glycoprotein, thus increasing the need for a meticulous study of its evolution using more sensitive bioinformatics tools [18] Extensive simulations have shown that analysis carried out with GARD [18], a method that employs a likelihood model selection procedure and searches multiple sequence alignments for evidence of recombination breakpoints [18,19], detect recombination events with higher power and accuracy than algorithms based on phylogeny discordance among large genomic segments [19].

We provide here an in depth evolutionary study of SARS-CoV-2 S glycoprotein using highly-sensitive phylogenetic recombination detection method [18,19] and natural selection analyses [20,21,22,23,24], paired with molecular (MD) [25] and essential (ED) [26] dynamic simulations to describe how the recombinant genetic makeup might have impacted the glycoprotein’s configuration [27,28]. We show that the recombination pattern of the SARS-CoV-2 S glycoprotein is far more complex than previously found, with four possible recombination events with potential ancestral viral lineages. The recombination evidence spans across SARS-CoV-2 S glycoprotein two subunits (S_1_ and S_2_), that share structural homology to its SARS-CoV-1 counterpart [29]. Subunit S_1_ includes the *C*-terminal domain 1/receptor binding domain (RBD) [30], which is responsible for binding to the angiotensin-converting enzyme 2 (ACE2) on the cell membrane, while subunit S_2_ plays a role during viral fusion with the cell membrane [31]. The RBD of each S monomer in the trimer complex, prior to binding to the target cell, is buried in the inactive “down” conformation (prefusion state) and cannot bind to ACE2 due to a steric clash [32]. In the process of virion-cell binding, one RBD monomer switches to a more exposed “up” conformation, inducing further concerted proteolytic cleavage events [33,34]. Because the recognition step between the RBD and the ACE2 is a dynamic process [35,36], it is fundamental to understand how these recombination events impacted the structural conformation of SARS-CoV-2 S glycoprotein altering or introducing novel structural constraints. To correlate the novel mosaic genomic makeup of SARS-CoV-2 with conformational changes in the S glycoprotein dynamics we employed MD simulations, a well-established technique for the characterization of biological macromolecules at atomic level, previously used for SARS-CoV-1 [27,28]. MD simulations were carried on SARS-CoV-2 S glycoprotein, as well as its bat and pangolin homologues, with the purpose of pinpointing similarities and differences between human and the animal reservoir glycoproteins; and understanding how the spatial structure of the S glycoprotein of the human isolate was shaped by its mosaic recombinant genotype. Our MD and ED analysis revealed not only that the major dynamic movements of SARS-CoV-2 were conserved despite recombination, but also that the up/down rotation of the RBD holds peculiar long-range covariance with key protein regions, such as the fusion peptide segment, the KR*SF cleavage site and, indirectly, with the newly acquired PRRA cleavage site. The comparison of the dynamic profile of the SARS-CoV-2 S glycoprotein to its homologues highlighted how the main dynamic features of SARS-CoV-2 S glycoprotein are shared among closely related CoVs strains circulating in wildlife. This finding was also corroborated by the strong purifying selection acting ancestrally to preserve key features of the novel variants, while indicating that conserved structural features adapted for efficient infection of multiple species. Our analysis further showed that the dynamic movements, essential for host cell binding, are maintained by conserved regions that we identified as two hinges. These conserved structural features will be useful for a rational design and development of panCoVs efficient inhibitors.

## 2. Results

### 2.1. Multiple Recombination Events and Purifying Selection Shaped SARS-CoV-2 S Glycoprotein

**Figure 1:**
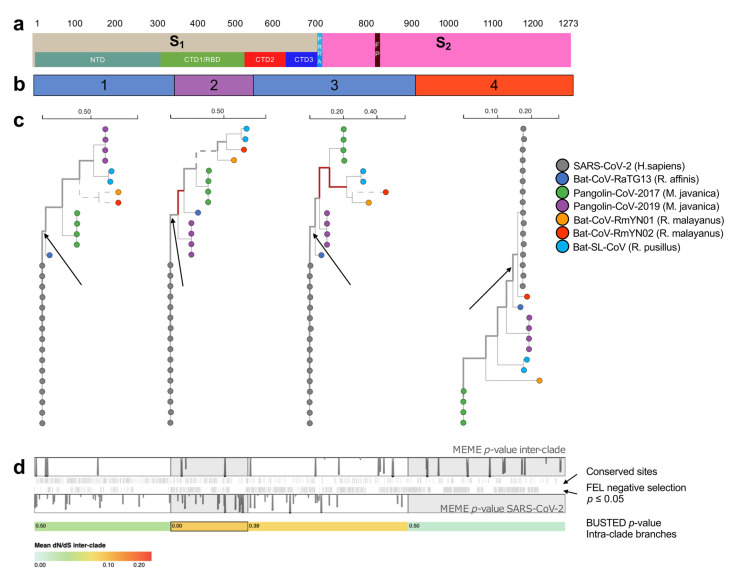
Selection analyses of S glycoprotein. Selection analyses were applied to a partitioned alignment of 29 CoV genomes. (**a**) Structure of the S gene. **S_1_**: subunit 1; **S_2_**; subunit 2; NTD: *N*-terminal domain; CTD1-3: *C*-terminal domain; RBD: receptor-binding domain; FP: fusion peptide. (**b**) Genetic fragments inferred at recombination analysis. The colors indicate the closest relative as shown by the maximum likelihood trees in panel c. (**c**) A maximum likelihood tree (rooting is arbitrary) for each genetic fragment is shown, and the inter-clade branches where host switch events might have occurred are indicated with thicker lines; branches where episodic selective pressure were detected (aBSREL *p* ≤ 0.05) are shown in red. Branches longer that 0.25 subs/site (under the MG94 codon model in aBSREL analyses) are censored at 0.25 subs/site and shown in dashed lines. (**d**) The impact of selective forces at individual sites is shown in two vertical bars at the top, where MEME *p*-values are shown either for the SARS-CoV-2 clade (bottom bar) or the inter-clade branches (top bar) as trail plots on top of each genetic fragment indicated here for simplicity by alternating white and grey rectangles. Tick-marks between the bars correspond to the location of sites that were inferred to be subject to pervasive purifying selection along the inter-clade branches, and sites where the amino-acid is conserved among all analyzed clades. The colored bar shows mean dN/dS along inter-clade branches (MG94 model) and the *p*-value for segment-wide episodic positive selection on the segment for intra-clade branches (BUSTED *p* ≤ 0.05)

Using sequences from public databases GISAID and NCBI (Supplementary Table S1), we investigated the evolutionary history of SARS-CoV-2 S glycoprotein utilizing the sensitive genetic algorithm GARD [18,19] (Figure 1). As compared to previous reports [14,15,37], our recombination analysis revealed that the S gene presents a complex mosaic genomic makeup composed of four genetic segments with different ancestral origin (Figure 1, Supplementary Table S2).

In order to clearly indicate where the recombinant breakpoints are located across the glycoprotein, we reported a schematic representation of the structural domains of the S gene (Figure 1a) above the representation of the recombinant genetic fragments (Figure 1b). Following the two panels, the first fragment comprises the whole *N*-terminal domain (NTD) (residues 14–303) and part of the RBD; the rest of this domain is included in the second fragment. The CTD2 spans across the second and third fragment, which also includes CTD3, the S_1_/S_2_ cleavage site and the fusion peptide (FP). The rest of the S_2_ subunit, including HR1 and HR2 regions, belong to the fourth fragment (Figure 1a,b).

The topology of the phylogenies resulting from each recombinant fragment (Figure 1c) revealed the evolutionary history of each genetic fragment (Figure 1b,c). In detail, the phylogenetic tree based on the fragment 1 and 3 showed that SARS-CoV-2 is closely related to Bat-CoV-RaTG13, isolated from the bat species *Rhinolophus affinis* in Yunnan province in China in 2013 [38]. This finding suggested that Bat-CoV-RaTG13 is the major recombinant parent of SARS-CoV-2 lineage. Based on these trees, and the signature pattern analysis (Supplementary Table S3, Supplementary Figure S1) that showed that SARS-CoV-2 residues in these fragments are similar to the ones in Bat-CoV-RaTG13, we conclude that these genetic fragments are compatible with the whole genome phylogeny [38], and likely part of the main SARS-CoV-2 genetic backbone, inherited from a Bat-CoV-RaTG13 ancestor (Figure 1c). Yet, we cannot exclude that this genetic information was acquired by recombination with another ancestral/unsampled strain circulating in bats, other than Bat-CoV-RaTG13.

The second fragment, containing the RBD (Figure 1a,b and Figure S1), was of recombinant origin. Its phylogeny indicated the Pangolin-CoV-2019 lineage as the closest relative to SARS-CoV-2 (Figure 1c). While the bootstrap support to this clade is not very strong, it is worth noting that the SARS-CoV-2 RBD is similar to the one found in Pangolin-CoV-2019 lineage, with only one amino acid difference (Supplementary Table S3, Supplementary Figure S1).

Finally, the phylogeny derived by the fragment 4 indicated Bat-CoV-RmYN02, obtained from bat species *R. malayanus* in Yunnan, China, in 2019, as the closest relative to SARS-CoV-2. Bat-CoV-RmYN02 presents insertions at the S_1_/S_2_ cleavage site similarly to SARS-CoV-2 [16]. As the PRRA insertions are found within the third genetic fragment, which does not share ancestry with Bat-CoV-RmYN02, it is possible that these residues have been independently acquired, as already proposed by Zhou et al. [16]. The close ancestral relationship, with strong bootstrap support, of SARS-CoV-2 with Bat-CoV-RmYN02 in the fourth genetic fragment further highlighted the chimeric nature of the S gene. In summary, the four potential recombinant genetic fragments marked by GARD revealed an underlying mosaic structure that was not reported previously for the S glycoprotein gene of SARS-CoV-2 (Figure 1b,c).

In order to determine selective pressure acting on the S gene, and because of the confounding impact of recombination on selection analyses [39,40,41], we analyzed each fragment, as partitioned by GARD (Figure 1b), independently (Figure 1d). We focused on internal branches to remove the biasing effects of unresolved intra-host evolution or sequencing errors [23]. A pattern of strong purifying selection (mean dN/dS = 0.019, 0.083, 0.072, 0.004 for each of the segments) was found on lineages that separate host clades forced evolution of the S glycoprotein (Figure 1d). A small fraction of residues (2%) second segment was found to be under strong episodic diversifying selection (dN/dS > 100, BUSTED *p* = 0.003). 412 variable residues out of 1057 were found to be evolving with dN/dS < 1 (*p*-value ≤ 0.05) along the intra-clade branches, including 76 residues out of 179 variable residues located in the RBD (Supplementary Table S4). There was evidence of episodic diversification in a few sites along the inter-clade branches (five sites with MEME *p* ≤ 0.05) and evidence of selection on several branches in GARD fragments 2 and 3 (not on the SARS-CoV-2 ancestral branch however). To identify sites which may be evolving adaptively in the SARS-CoV-2 clade, we tested for episodic diversifying selection on all branches in this clade (this is likely too permissive, but we wish to err on the side of additional power) and identified fourteen such sites, eight in S_1_ (*p* ≤ 0.05) (Figure 1, Supplementary Table S5).

These analyses confirmed that strong purifying selection predominantly constrained the variability of the residues during host switching. In presence of extensive recombination, strong purifying selection is not surprising, and it has been described previously for the genomes of other RNA recombinant viruses [9]. While mutation is an evolutionary mechanism for any genome, there can be local fitness optima which might limit the adaptability of viruses to new hosts [42,43], and the proofreading and mismatch correction system coded by CoV genomes reduces their mutation rates when compared to other RNA viruses [3,4,5]. Recombination is an important drive in CoVs evolution [2,8]. The lack of positive selection in our results, together with the multiple ancestry of the S gene, confirms that the current genotype harboring the RBD and the newly acquired furin-like cleavage site [44] were most likely acquired as a result of recombination events from unknown ancestors. This supports the hypothesis that the genetic configuration of SARS-CoV-2, able to effectively spread within the human population, was reached in the animal host, prior to jump to humans [44].

### 2.2. Long-Range Correlated Domain Motions are Common between SARS-CoV-2 and Ancestral CoVs of Bat and Pangolin

We investigated the impact of recombination on the structural dynamics of the S glycoprotein systems of SARS-CoV-2, Bat-CoV-RaTG13 and Pangolin-CoV-2017. We first explored dynamic similarity of the three systems by comparing the per-residue root mean square fluctuations (RMSF) observed during the 630 ns of the respective simulations (Supplementary Figure S2). The results revealed noteworthy differences between the three S monomers within the same system. Monomer 3 (in green in Supplementary Figure S2) in SARS-CoV-2 shows higher fluctuations in the NTD, while monomer 1 (in blue in Supplementary Figure S2), the one assuming the “up” conformation during the simulation, is more mobile in the RBD (Supplementary Figure S2a). Monomer 2 (in magenta in Supplementary Figure S2) shows the highest absolute RMSF around residue 250 in NTD and residues 624–629 (within recombinant segment 4). Peaks in fluctuations in residues 144–153 (within recombinant segment 1), 624–629 (within recombinant segment 4), and 834–847 (within recombinant segment 5) are of interest for the protein long-range communication, discussed in depth below. Differences in fluctuations among the three monomers are also observed in Bat-CoV-RaTG13 glycoprotein (Supplementary Figure S2b), although in this case the same monomer 1 shows the highest fluctuations in both NTD and RBD. The Pangolin-CoV-2017 S glycoprotein shows instead the lowest global fluctuations, with small differences among monomers (Supplementary Figure S2c).

**Figure 2:**
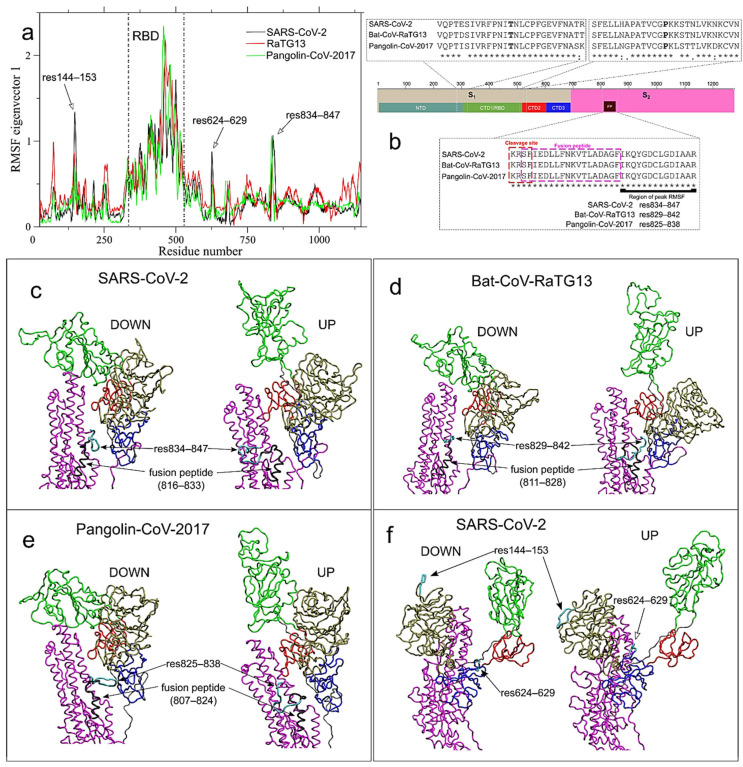
Long-range correlated motions along ED eigenvector 1. (**a**) Per-residue Root Mean Square Fluctuations (RMSF) in nm of the S glycoprotein filtered trajectory along ED eigenvectors 1 for SARS-CoV-2, Bat-CoV-RaTG13, and Pangolin-CoV are reported in black, red and green colors, respectively. Residues corresponding to peak of fluctuations in SARS-CoV-2 are reported. (**b**) Local alignment in the conserved regions of hinge 1 (Thr333), hinge 2 (Pro527) and fusion peptide (residues 816–833). In the alignment subsets, conserved residues are marked by “*”; “.” indicates that one strain is different from SARS-CoV-2 at the marked position; “:” indicates both other strains differ from SARS-CoV-2. (**c**–**e**) The extreme conformations of the S protein MD trajectories along Essential Dynamics (ED) eigenvector 1 for SARS-CoV-2, Bat-CoV-RaTG13 and Pangolin-CoV-2017 are shown in panel c, d and e, respectively (see also Supplementary movies S1–S3). The three portions of the S_1_ subunit (RBD, CTD2, and CTD3) are shown in green, red, and blue, respectively. The NTD and S_2_ subunit are in tan and magenta, respectively. The two extreme conformations correspond to the up and down RBD conformations and are indicated as DOWN and UP in the left and right panels, respectively. Residues 834–847 and the adjacent fusion peptide are highlighted in cyan and black colors, respectively for SARS-CoV-2. (**c**). The same conserved regions in Bat-CoV-RaTG13 and Pangolin-CoV-2017 are indicated in corresponding panels (**d**) and (**e**). Animations of the correlated motion between RBD and res834–847, adjacent to the fusion peptide, are shown in Supplementary movies S4–S6 for SARS-CoV-2, Bat-CoV-RaTG13 and Pangolin- CoV-2017, respectively. (**f**) Projection of the S protein MD trajectory along ED eigenvector 1 for the SARS-CoV-2 system. Residues 144–153 in NTD and 624–629 in CTD3, corresponding to peak of fluctuations shown in panel (**a**), are shown in cyan color.

**Figure 3:**
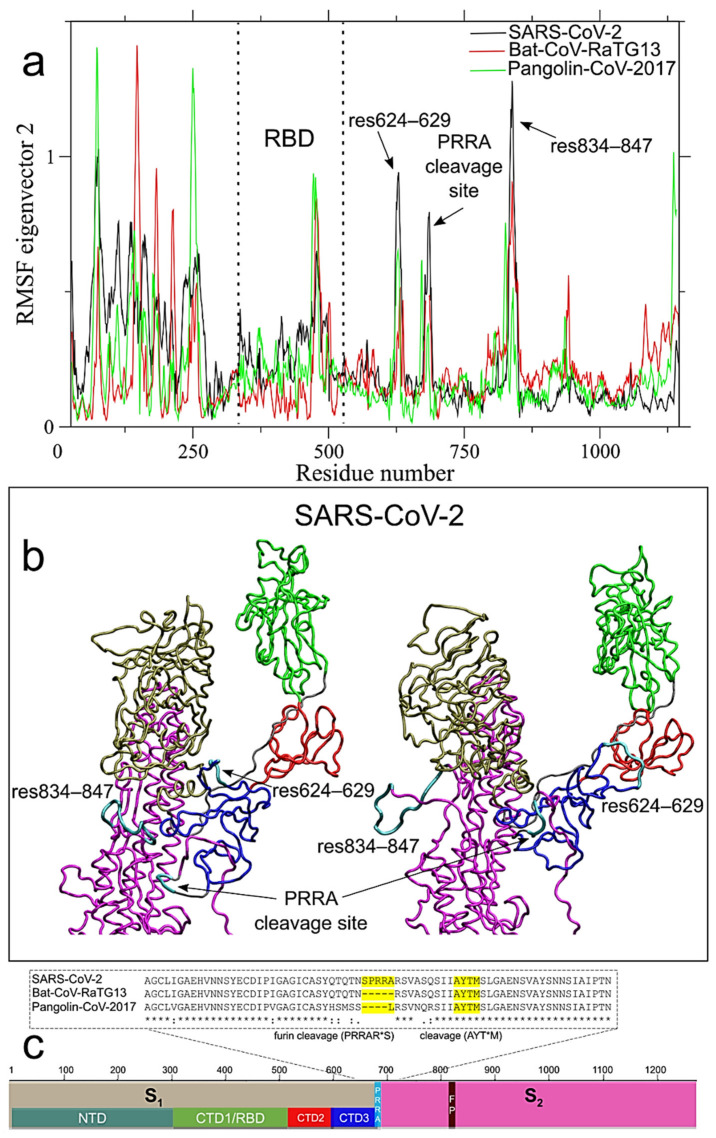
Long-range correlated motions along ED eigenvector 2. (**a**) Per-residue Root Mean Square Fluctuations (RMSF) in nm of the S protein filtered trajectory along ED eigenvectors 2 for SARS-CoV-2, Bat-CoV-RaTG13 and Pangolin-CoV-2017 are reported in black, red and green colors, respectively. Residues corresponding to peak of fluctuations in SARS-CoV-2 are reported. (**b**) Projection of the S protein MD trajectory along ED eigenvector 2 for the SARS-CoV-2 system. Residues 624–629, the newly acquired PRRA cleavage site and res834–847, corresponding to peak of fluctuations shown in panel (**a**), are highlighted in cyan color. (**c**) Local alignment in the PRRA cleavage site. In the alignment subsets, conserved residues are marked by “*”; “.” indicates that one strain is different from SARS-CoV-2 at the marked position; “:” indicates both other strains differ from SARS-CoV-2.

Notwithstanding the informativeness of the RMSF analysis, the total fluctuations are not ideal to identify the long-range communications needed by the S glycoprotein to coordinate the action of receptor-binding and proteolytic processing for virus-cell fusion. Protein fluctuations, in fact, can be divided in “small uninteresting motions”, which are uncorrelated with other protein motions, and “large collective protein movements”, connected to functional properties. We therefore sought to detect the latter employing the ED technique [26]. ED analysis is based on the diagonalization of the covariance matrix built from the atomic fluctuations after the removal of the translational and rotational movement [26]. It is usually applied only on the c-alpha atoms, since they describe the motion of the protein main chain [26]. Therefore, eigenvectors associated with the largest eigenvalues of the matrix represent a large fraction of the total protein motion, and the projection of the trajectory along these first eigenvectors highlights the large collective protein movements [26]. Since our main interest is focused on the conformational basin of the S glycoprotein—available conformations visited by the S protein—rather than on the interaction in the trimer, we concatenated the production trajectory of each monomer to produce a total 1875 ns long trajectory for each protein. The resulting trajectories contain all the conformations visited by the three S glycoproteins, which were analyzed with ED technique and plot of filtered RMSF along the first eigenvectors (i.e., the ones with the largest eigenvalues) identify highly correlated protein movements. Figure 2a shows the filtered RMSF along eigenvector 1, capturing 58.8% of the total protein motion in SARS-CoV-2 (black line in Figure 2a). Eigenvector 2 captures 26.1% of total motion in SARS-CoV-2; the other 3360 eigenvectors contained the remaining 15.4% of the global motion, with the third eigenvector coming in at 2.2% of the total motion. Therefore, our simulations effectively separated the large collective global motion from the small uninteresting fluctuations. Projections onto the primary eigenvector of the animal counterparts of SARS-CoV-2, Bat-CoV-RaTG13 and Pangolin-CoV-2017 S glycoproteins (red and green lines in Figure 2a, respectively), contained 68.4% and 64.8% of the total protein motion, respectively. While the global RMSF are quite different in the three systems (Supplementary Figure S2); the filtered RMSFs, along eigenvector 1, are very similar (compare black, red and green lines in Figure 2a), indicating that the major long-range correlated motions are shared among the three proteins. The comparison of RMSF filtered along eigenvector 1–2 against the global RMSF highlighted the role of different glycoprotein regions in SARS-CoV-2 glycoprotein. The highest RMSF in SARS-CoV-2 is located in the NTD, around residue 250—first genetic fragment (Supplementary Figure S2a), but that peak is completely absent in eigenvector 1 (black line in Figure 2a) or eigenvector 2 (black line in Figure 3a), while only a peak in residues 144–153 is observed along eigenvector 1. Therefore, fluctuations of the region around 250, found in a region that likely did not undergo recombination, have a random uncorrelated character and do not play a role in long-range communications in the pre-fusion conformation of the S glycoprotein.

ED analysis revealed that, independently of recombination, RBD region shows the most correlated protein motion (Figure 2a). The motions can be appreciated by the visualization of the two extreme configurations along eigenvector 1 (Supplementary Figure S3a,c for SARS-CoV-2, Bat-CoV-RaTG13 and Pangolin-CoV-2017, respectively; Supplementary Movies S1–S3). Although it is not surprising that RBD rotation in the opening/closing mechanism, functional to receptor recognition, is the most correlated motion in S glycoproteins among all lineages, these simulations revealed a new piece of information: the presence of two amino acid hinges that structurally separate the RBD by the NTD (hinge 1) and CTD2 (hinge 2), and that are found at the core of the open/close described movement. Hinge 1 is located around residue Thr333, while hinge 2 is located around Pro527, both in fragment 2. The *N*- and *C*-terminal regions of CTD1, where the two hinges are located, are conserved (see Figure 2b, with the two hinges highlighted in bold). Another proline residue (Pro330) is present in the hinge 1 region of SARS-CoV-2 S glycoprotein and may play an important role in dictating protein structure by restricting its backbone conformation (54). Both Pro330 and Pro527 are under negative selection (Supplementary Table S4), and are functional to the observed rotation mechanism that regulates the interaction between the S glycoprotein and the host receptor, a common feature among the three systems.

Another dynamic and novel feature captured by our simulations, common to SARS-CoV-2, Bat-CoV-RaTG13 and Pangolin-CoV-2017 S glycoproteins, is a fluctuation peak involving residues 834–847 (one of which is under negative selection, and several have border-line *p*-values—Supplementary Table S4) and located at the C-term of the FP and the S_2_ cleavage site KR*SF [45] of SARS-CoV-2 (within the fragment 3, see Figure 2b). This peak is also present in the other two systems along eigenvector 1, although to a lesser extent in the Bat-CoV-RaTG13 (red line in Figure 2a). This conserved region, may be critical to the long-range protein communication needed by the S glycoprotein to orchestrate the different cleavage steps. The extreme conformations along eigenvector 1, visited by residues 834–847 in SARS-CoV-2, are shown in Figure 2c (cyan region), together with the FP immediately at their *N*-terminal (black residues). The corresponding regions in the Bat-CoV-RaTG13 and Pangolin-CoV-2017 S glycoproteins are shown in Figure 2d,e, respectively. The complete motion of these regions in the three systems is represented for SARS-CoV-2, Bat-CoV-RaTG13 and Pangolin-CoV-2017, in Supplementary movies S4–S6, respectively. The finding that the conserved correlated movements in SARS-CoV-2 S glycoprotein span across fragments potentially originated from different ancestors is in agreement with strong purifying selection acting across lineages, likely preserving the biological relevant functional protein motions in both human and animal CoVs.

### 2.3. Long-Range Correlated Motions Peculiar of SARS-CoV-2 Involve the Newly Acquired Furin-Like Cleavage Site

The majority of the residues that differentiate SARS-CoV-2 S glycoprotein from the Bat-CoV-RaTG13 or Pangolin-CoV-2017 are found within the NTD portion of the S_1_ subunit (Supplementary Figure S1, Supplementary Table S5). The uniqueness of SARS-CoV-2 S glycoprotein is reflected by the different RMSF profile of NTD along ED eigenvector 2 (see Figure 3a). The S glycoprotein of SARS-CoV-2 presents a conserved cleavage site (KR*SF) located at the C-term of the FP (fragment 3) and the S_2_ [45], shown in Figure 2b, and a unique furin-like cleavage sequence (PRRARS*V) in positions 681–684 in fragment 3 as well, (Figure 3c).

While the general profile of RMSF along eigenvector 1 is quite conserved across the three systems, two high RMSF peaks are found only in SARS-CoV-2 around residues 144–153 (within the fragment 1) in NTD; and residues 624–629 (fragment 3) in CTD3 (see Figure 2a). The first peak is the only one in NTD higher than 1 nm along this eigenvector (1.34 nm in SARS-CoV-2 vs only 0.86 and 0.70 in Bat-CoV-RaTG13 and Pangolin-CoV-2017, respectively), while the second peak (residues 834–847) is at the *C*-terminal of the FP and the S_2_ cleavage site (KR*SF), within the fragment 3 (see Figure 2b). The extreme conformations along eigenvector 1, visited by residues 144–153 and 624–629 in SARS-CoV-2, are highlighted in Figure 2f. This long-range correlated motion involving regions of NTD, CTD3 and RBD likely reflect the cooperative character of the interaction between the RBD of one monomer and the other monomers, whose conformations could help in the regulation of the subsequent steps of fusion with the host cell membrane [34]. This hypothesis is reinforced by the correlated motion of the same residues 624–629 region, with the newly acquired furin-like cleavage site region (residues 681–684) and residues 834–847 along eigenvector 2, that are not observed in Bat-CoV-RaTG13 and Pangolin-CoV-2017 (see Figure 3a). The two extreme projections of the SARS-CoV-2 MD trajectory along ED eigenvector 2 are shown in Figure 3b, with the three mentioned regions in cyan color, while the complete motion is reported in Supplementary movie S7.

### 2.4. Long-Range Correlated Motions Conserved in the Glycosylated SARS-CoV-2

Glycans hold an important role in the shielding of peptides epitopes of S glycoprotein and in the modulation of its interactions with the ACE2 [46]. Furthermore, MD simulations and experimental results showed that the glycans at site N165 and N234 play an essential structural role in modulating the dynamics of RBD in “up” state [47]. Therefore, we investigated whether the evidenced correlated long-range movements in SARS-CoV-2 are also conserved in the glycosylated model of the S protein. Supplementary Figure S4 shows the comparison of RMSF in the non-glycosylated (Supplementary Figure S4a) and glycosylated S protein RMSF (Supplementary Figure S4b). The glycosylation produces a general reduction in protein flexibility, but all the peaks in key regions discussed above, including the RBD and the furin-like cleavage site, are present.

**Figure 4:**
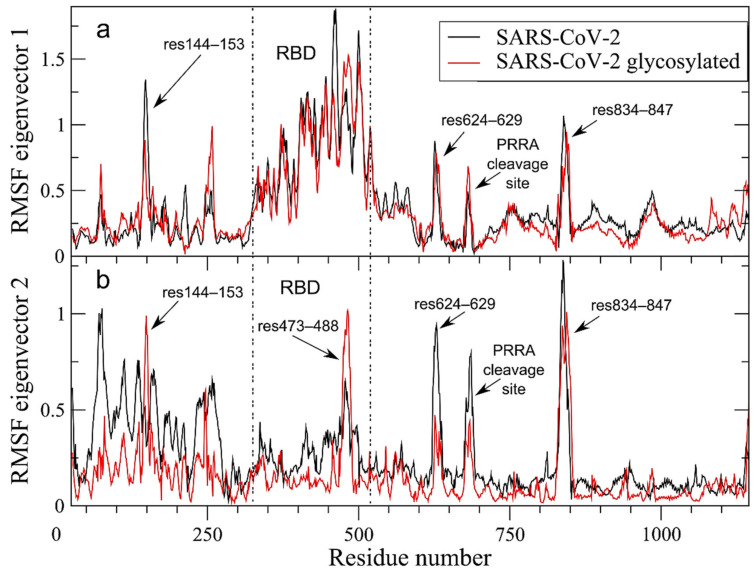
Per-residue Root Mean Square Fluctuations (RMSF) in nm of the S glycoprotein filtered trajectory along ED eigenvectors 1 (panel **a**) and 2 (panel **b**) for SARS-CoV-2, unglycosylated and glycosylated forms are reported in black and red colors, respectively.

Filtering of large collective protein movements through ED analysis shows a striking similarity of fluctuations along eigenvector 1 between unglycosylated (black line in Figure 4a) and glycosylated S protein RMSF (red line in Figure 4a). Projection of the two extreme configurations along ED eigenvector 1 is shown in Supplementary Figure S5 for the two systems. The conservation of the rotation of RBD in up/down conformation is evident and the motion along eigenvector 1 accounts for 58.8% and 70.4% of the total protein motion for the unglycosylated and glycosylated systems, respectively. The RMSF profile along eigenvector 2 is conserved in RBD and S2 (Figure 4b), with peaks of fluctuations differing only in height but not in position. It is worth mentioning that i) the peak in NTD involving 144–153 is reduced in the glycosylated form along eigenvector 1 (Figure 4a) but it is increased along eigenvector 2, confirming the importance of this region in the S protein dynamics; ii) the peak in RBD in the glycosylated system, along eigenvector 2 (Figure 4b) is centered on residues 473–488, i.e., a region that directly interacts with the human ACE2 receptor [48].

In summary, our analyses highlighted the complex network of long-range correlated motions in SARS-CoV-2 compared to the bat and pangolin counterparts, involving i) the PRRA cleavage site (residues 681–684); ii) the KR*SF cleavage site (residues 834–847), close to the fusion peptide region; iii) residues 624–629 in CTD3 (Figure 3). The last two regions are in turn connected to iv) the RBD rotation (Figure 2c) and v) residues 144–153 in NTD (Figure 2f). This network is maintained in the glycosylated form (Figure 4). Although some of these features are also observed in Bat-CoV-RaTG13 and Pangolin-CoV-2017 strains, the strain circulating in human clearly shows an increase of long-range correlated motions, likely acquired through mosaic recombination.

## 3. Discussion

Recombination is a hallmark of CoV evolution [8]. Our findings revealed a mosaic nature of the SARS-CoV-2 S glycoprotein that was underappreciated to date [15,37]. We found evidence that several recombination events occurred, involving ancestral Pangolin-CoV-2019, Bat-CoV-RaTG13, and Bat-CoV-RmYN02 lineages; however, the limited sampling of CoVs in wildlife may complicate the definitive inference of the parental lineage of each fragment. Even for genetic segments such as the RBD, where there is a high similarity between Pangolin-CoV-2019 and SARS-CoV-2, the closest relative to the pandemic strain may actually be among the vast unsampled population circulating in bats, and the pangolin sequence that we have knowledge of, so far, may not be the direct ancestor. Extensive genomic surveillance of CoVs in wildlife is needed to ascertain the events that shaped the current spike mosaic gene configuration. In summary, our analyses suggest that the genetic makeup contributing to enhanced dynamic movements of SARS-CoV-2 S glycoprotein was mostly acquired through recombination, while only occasionally from point mutations.

One of the recombination events led to the acquisition of the ACE2 binding residues that are found in the currently circulating SARS-CoV-2 lineage. The novel furin-like cleavage in the S_1_/S_2_ junction (PRRARS*V, residues 681–684) is also found within one of the potential recombination fragments [44]. This new feature of SARS-CoV-2 was not present in the other closely related CoVs [49,50] or previously circulating SARS-CoVs [44], and our analysis confirmed that it was likely acquired independently from the recently reported RmYN02 strain. Hence, the suggestion that recombination allowed SARS-CoV-2 to gain the current configuration and the ability to make such a successful species jump to humans [44]. Moreover, absence of detectable positive diversifying selection on the branch that leads to the recent human CoV-2 lineage, together with the strong purifying negative selection exerted on the majority of the glycoprotein, is compatible with the hypothesis that recombination might have led to the ability to efficiently infect humans, rather than selection within the human epidemic. Such a scenario highlights the necessity of active surveillance wherever there are interactions at risk between wildlife, livestock, and humans.

Molecular and essential dynamics simulations provided insight on how recombination affected the functionality of the S glycoprotein by surprisingly enhancing its long-range correlated fluctuations, fundamental to its activity. Further, our simulations revealed the presence of a complex network of long-range correlated motions among key regions in SARS-CoV-2 S glycoprotein, such as the RBD, the furin-like cleavage site and the fusion peptide. These long-range correlated movements are fundamental for the effective two-step sequential protease cleavage, as well as for the activation of CoV S glycoproteins, as shown for SARS-CoV and MERS-CoV [34,51]. Our analyses indicated that these regions containing the residues necessary to maintain the long co-variant movements of SARS-CoV-2 S glycoprotein were conserved as a result of undergoing strong negative selection.

We also identified structural and dynamic features that are preserved among human and ancestral glycoprotein, and that may be targeted for the development of panCoVs efficient inhibitors. These included the rotation of RBD in up/down conformation, and the two regions that separate RBD from NTD and CTD2 and act as a hinge in the opening/closing mechanism. Small molecules targeting these regions and inhibiting such motions may reduce efficient binding of SARS-CoV-2 glycoprotein to the host cell membrane.

In summary, our analyses described the duality of purifying selection, acting as a conservation mechanism to preserve fundamental biological activity of the S glycoprotein, and recombination, which puzzled together genetic pieces that showed increased conformational dynamicity of SARS-CoV-2 S glycoprotein. Such a concerted machinery dictated the emergence of a novel glycoprotein able to establish more efficient interactions with the host cell surface receptor. We conclude that the dynamic covariance was preserved, despite the pervasive recombination history of SARS-CoV-2 S glycoprotein, by the action of strong purifying selection, occurring in the animal reservoir prior to the jump to human.

## 4. Materials and Methods

### 4.1. Genetic Data

We initially selected SARS-CoV-2 and closely related Beta-CoVs genomic sequences from GISAID and NCBI (Supplementary Table S1), for a total of 133 isolates. We downloaded the genomes of SARS-CoV-2 strains from December 2019 until March 2020, and added related bat and pangolin viruses available in GISAID on 20 November 2020 for which at least 70% of the S gene was sequenced. This initial set was later reduced as explained in the recombination and selection methods. Genome sequences were aligned using MAFFT [52] and refined manually. Alignments for the glycoprotein were extracted from the genome alignment.

We referred to the CoV RaTG13 strain (NCBI accession no. MN996532; GISAID id EPI_ISL_402131), isolated from bats in the Yunnan province, China, in 2013 as Bat-CoV-RaTG13; and to the CoV lineages isolated from pangolins in China in 2017 (EPI_ISL_410538-43) and 2019 (EPI_ISL_412860 and EPI_ISL_410721) as Pangolin-CoV-2017 and Pangolin-CoV-2019, respectively. We used the strain names Bat-CoV-RmYN01 and Bat-CoV-RmYN02 to indicate the bat CoVs EPI_ISL_412976 and EPI_ISL_412977, isolated from Yunnan, China, in 2019. We referred to the bat SARS-like CoVs strains MG772933 and MG772934 isolated in China, respectively in 2017 and 2015, as Bat-SL-CoVs.

### 4.2. Recombination and Selection Analyses

Recombination analysis was performed using the genetic algorithm for recombination detection, GARD [18]. Since GARD loses power when there are many closely related sequences, and at present there is no evidence of ongoing recombination in SARS-CoV-2 isolates, the dataset was reduced by clustering isolates which differed by 0.1% or less, reducing the number of sequences to 29 (Supplementary Table S1). Maximum likelihood (ML) trees were calculated separately for each segment using raxml-ng and the GTR+G+I model with 5 random parsimony starting trees. Within these trees, we further identified three branches that included host-switching events and the branch separating Pangolin-CoV-2017 and Pangolin-CoV-2019 isolates. We used BUSTED [20] to assess the presence of episodic diversifying selection on the gene S partitions. FEL [21] and MEME [22] methods were used on this partitioned alignment to look for pervasive (FEL) or episodic (MEME) diversifying positive selection affecting the four inter-clade branches, and, in a separate analysis, affecting only the SARS-CoV-2 clade. To look for lineage specific selection on inter-clade branches we used the aBSREL [23] method separately on each partition (since it cannot be applied to multi-partition data). All selection analyses were carried out in HyPhy v2.5.14 [24].

### 4.3. Structure Homology Modeling

S glycoprotein amino acid sequences of human SARS-CoV-2 Wuhan-Hu-1 (Accession no. QHD43416), Bat-CoV-RaTG13, and Pangolin-CoV-2017 (EPI_ISL_410539) were submitted to Swiss-model [53]. The best models (highest values of Global Model Quality Estimation [GMQE]) were selected and used as templates. Alignments of targets and templates amino acid sequences were performed using EMBOSS Needle, with default score matrix BLOSUM 62 [54]. The 3D homology homo-trimer models were created with the Modeller v9. 23 automodel class [55,56] using the alignment as a guide, followed by Discrete Optimized Protein Energy Score (DOPE) based model selection [57] and refinements were conducted using Modeller v9.23 scripts. Final structure model validation was conducted with ProSA [58,59] and QMEAN servers [60,61]. Visualizations of the atomic models, including figures and movies, were created with Chimera v1.12 [62] and VMD v1.9.2 [63]. The VESPA algorithm [64] was used to identify new features of SARS-CoV-2 as compared to pangolin and bat lineages; the SARS-CoV-2 Wuhan-Hu-1 strain (Accession MN908947.3) was used as reference for the codon coordinates. The glycosylated form of the human SARS-CoV-2 was also built; an asymmetric glycosylation of the three protomers has been derived by glycoanalyitic data for the N-glycans [65] and O-glycans [66] according to the work of Casalino et al. [47]. The proteins were modeled using Amber14SB force field [67] and the carbohydrate moieties by the GLYCAM06j-1 version of GLYCAM06 force field [68].

### 4.4. Molecular Dynamics Simulation

Structures of human SARS-CoV-2, Bat-CoV-RaTG13, and Pangolin-CoV-2017, obtained for homology modeling, were used as starting points for MD simulations. Topology files were generated with the pdb2gmx GROMACS tool, using the amber99sb forcefield [25]. Proteins were embedded in a triclinic box, extending up to 15 Å from the solute, and immersed in TIP3P water molecules [69]. Counter ions were added to neutralize the overall charge with the genion GROMACS tool. After energy minimizations, the systems were slowly relaxed for 5 ns by applying positional restraints of 1000 kJ mol^−1^ nm-2 to the protein atoms. Following this step, unrestrained MD simulations were carried out for a length of 630 ns for each system, with a time step of 2 fs, using GROMACS 2018.3 simulation package (supercomputer Galileo and Marconi-100, CINECA, Bologna, Italy) [70]. V-rescale temperature coupling was employed to keep the temperature constant at 300 K [71]. The Particle-Mesh Ewald method was used for the treatment of the long-range electrostatic interactions [72]. The first 5 ns of each trajectory were excluded from the analysis.

The same MD protocol has been applied to the glycosylated model of the SARS-CoV-2 S trimer, generated using the glycoprotein builder available at GLYCAM-Web (www.glycam.org).

We carried out the ED analysis to identify the main 3D directions along which the majority of the protein motion is defined [26]. We concatenated the production trajectory of each monomer to produce a total 1875 ns long trajectory containing all the conformations visited by each S proteins, that was analyzed with ED using the GROMACS covar and anaeig tools. Main principal component movements were checked to be conserved in different time windows. RMSF was calculated with the GROMACS rms tool.

## Data Availability

Publicly available datasets were analyzed in this study. This data can be found here: https://www.gisaid.org/ and https://www.ncbi.nlm.nih.gov/nuccore.

## Supplementary Materials

The following are available online at https://www.mdpi.com/1422-0067/22/1/80/s1.

## Glossary

CoV	Coronavirus
COVID-19	Coronavirus disease 2019
SARS	Severe acute respiratory syndrome
SARS-CoV-2	Severe acute respiratory syndrome coronavirus 2
Bat-CoV-RaTG13	Bat coronavirus RaTG13
Pangolin-CoV-2017	Pangolin coronavirus 2017
Pangolin-CoV-2019	Pangolin coronavirus 2019
Bat-CoV-RmYN01	Bat coronavirus RmYN01
Bat-CoV-RmYN02	Bat coronavirus RmYN02
Bat-SL-CoV	Bat SARS-like coronavirus
RBD	Receptor binding domain
NTD	*N*-terminal domain
CTD1	*C*-terminal domain 1
CTD2	*C*-terminal domain 2
CTD3	*C*-terminal domain 3
MD	Molecular Dynamics
ED	Essential dynamics
ACE2	Angiotensin-Converting Enzyme 2
FP	Fusion peptide
RMSF	Root Mean Square Fluctuations
