# Supplementary material for: Multiple Recombination Events and Strong Purifying Selection at the Origin of SARS-CoV-2 Spike Glycoprotein Increased Correlated Dynamic Movements
Source: Int J Mol Sci. 2020 Dec 23;22(1):80. doi: 10.3390/ijms22010080 (PMC7794730; doi:10.3390/ijms22010080)
Supplement: Supplementary file 1 [file ijms-22-00080-s001.zip › Supplementary.Figures.pdf]

Supplementary Figures

Multiple Recombination events and Strong Purifying Selection At The Origin Of SARS-CoV-2 Spike Glycoprotein Increased Correlated Dynamic Movements

Massimiliano S. Tagliamonte [1,2,#], Nabil Abid [3,4#], Stefano Borocci [5,6], Elisa Sangiovanni [5], David A. Ostrov [2], Sergei L. Kosakovsky Pond [7], Marco Salemi [1,2,*], Giovanni Chillemi [5,8,*,$] and Carla Mavian [1,2,*,$]

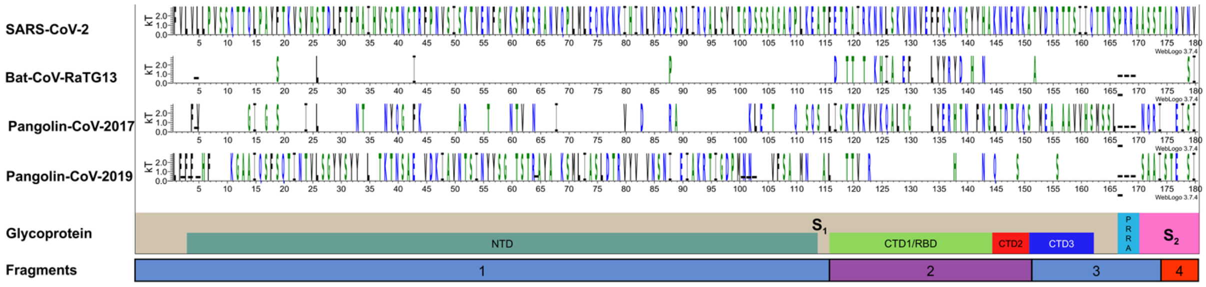

Figure S1. Signature residues in the S glycoprotein of SARS-CoV-2. Signature residues that distinguish human SARS-COV-2 from closely related bat and pangolin (lineage isolated in 2017 in China and lineage isolated in 2019 in China) isolates; residues are colored according their charge: hydrophilic (RKDENQ) residues in blue, neutral (SGHTAP) in green, and hydrophobic (YVMCLFIW) in black. Numbers correspond to the residue position in Wuhan-Hu-1 isolate (NCBI accession number MN908947.3). In the box below we show a schematic representation of the spike glycoprotein is given: S1 and the S2 subunits of the S glycoprotein are indicated in grey and fuchsia, while CTD1/RBD is shown green, CTD2 in red, CTD3 in blue. The newly acquired S1/S2 cleavage site “PRRA” is also indicated in cyan. Recombinant fragments are shown at the bottom as indicated in Figure 1.

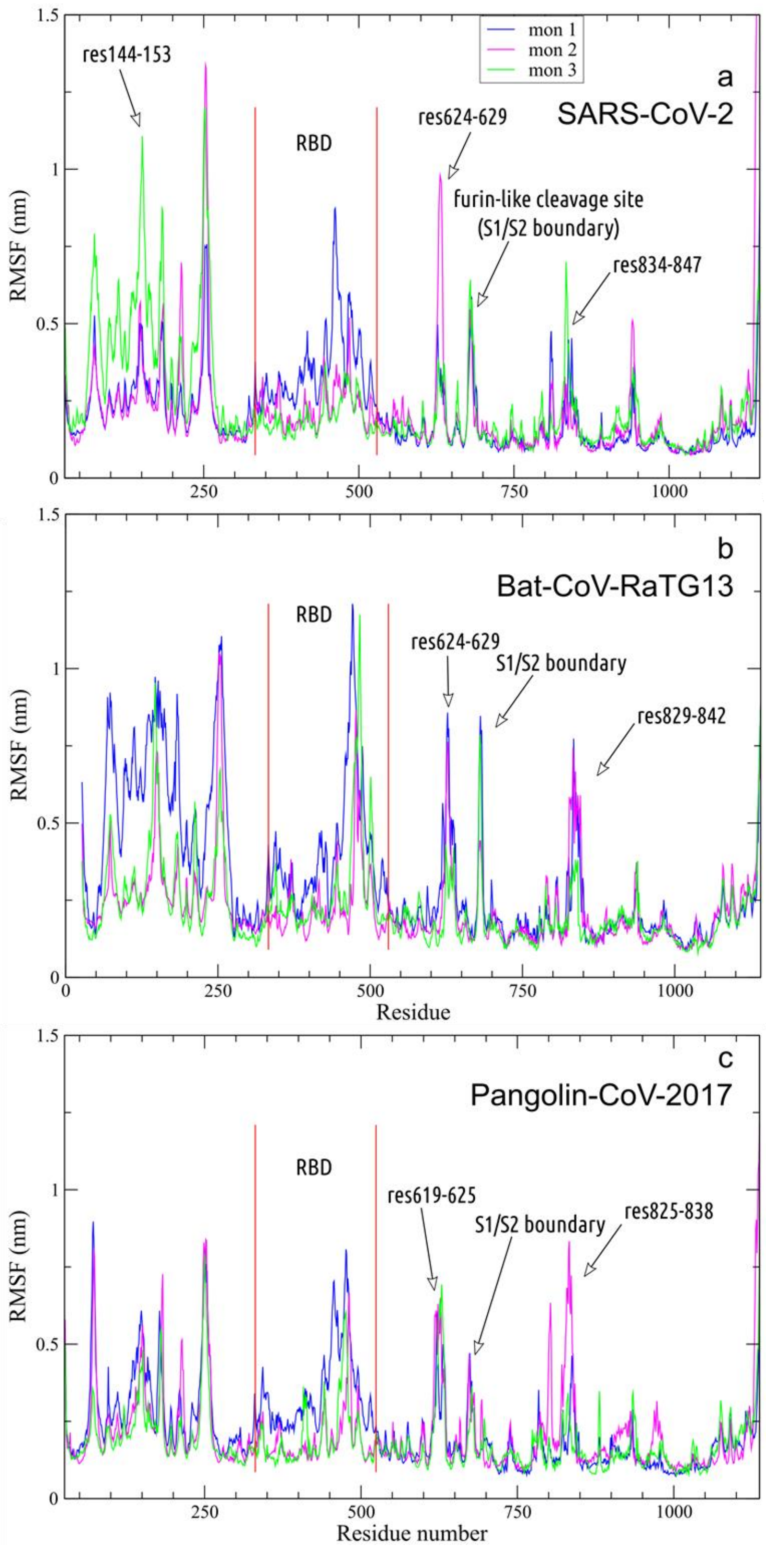

Figure S2. Per-residue RMSF of SARS-CoV-2, Bat-CoV-RaTG13 and Pangolin-CoV-2017 S protein are shown in panels a, b and c, respectively. The three S monomers are colored in blue, magenta and green, respectively. SARS-CoV-2 peak of fluctuations in CTD3 (res624-629), at the S1/S2 cleavage (where SARS-CoV-2 has the newly acquired furin-like cleavage site) and in S2 (res834-847) are observed in corresponding residues of the two other systems.

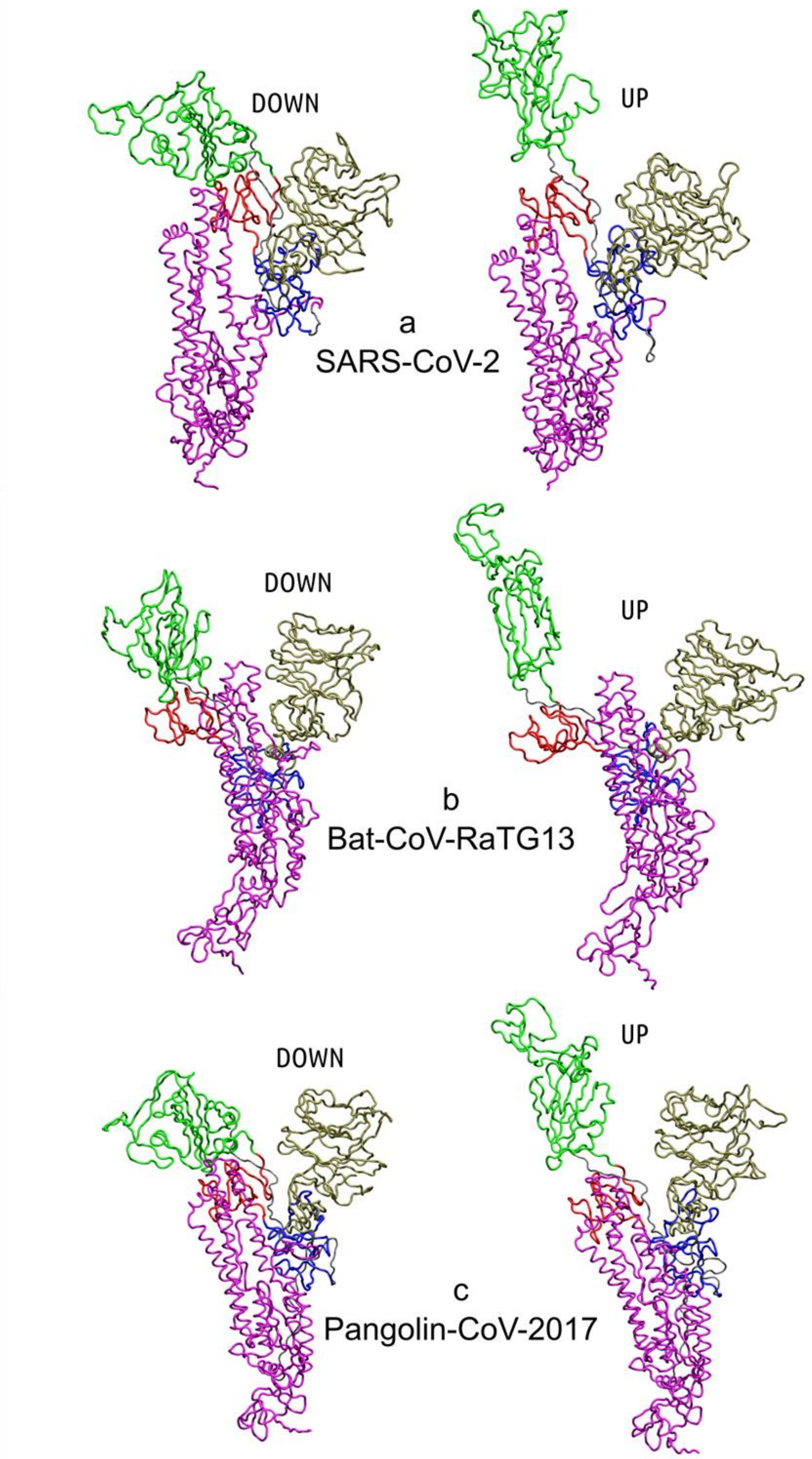

Figure S3. Projection of the two extreme configurations for the S protein MD trajectories along Essential Dynamics (ED) eigenvector 1 for SARS-CoV-2 (a), Bat-CoV-RaTG13 (b) and Pangolin-CoV-2017 (c). The three portions of the S1 subunit (RBD, CTD2, and CTD3) are shown in green, red, and blue colors, respectively. The NTD and S2 subunit are in tan and magenta colors, respectively. The two extreme configurations correspond to the up and down RBD conformations and are indicated as DOWN and UP in the left and right panels, respectively. The up/down rotation of RBD is the dominant correlated motion in all three SARS-CoVs. See also Supplementary movies.

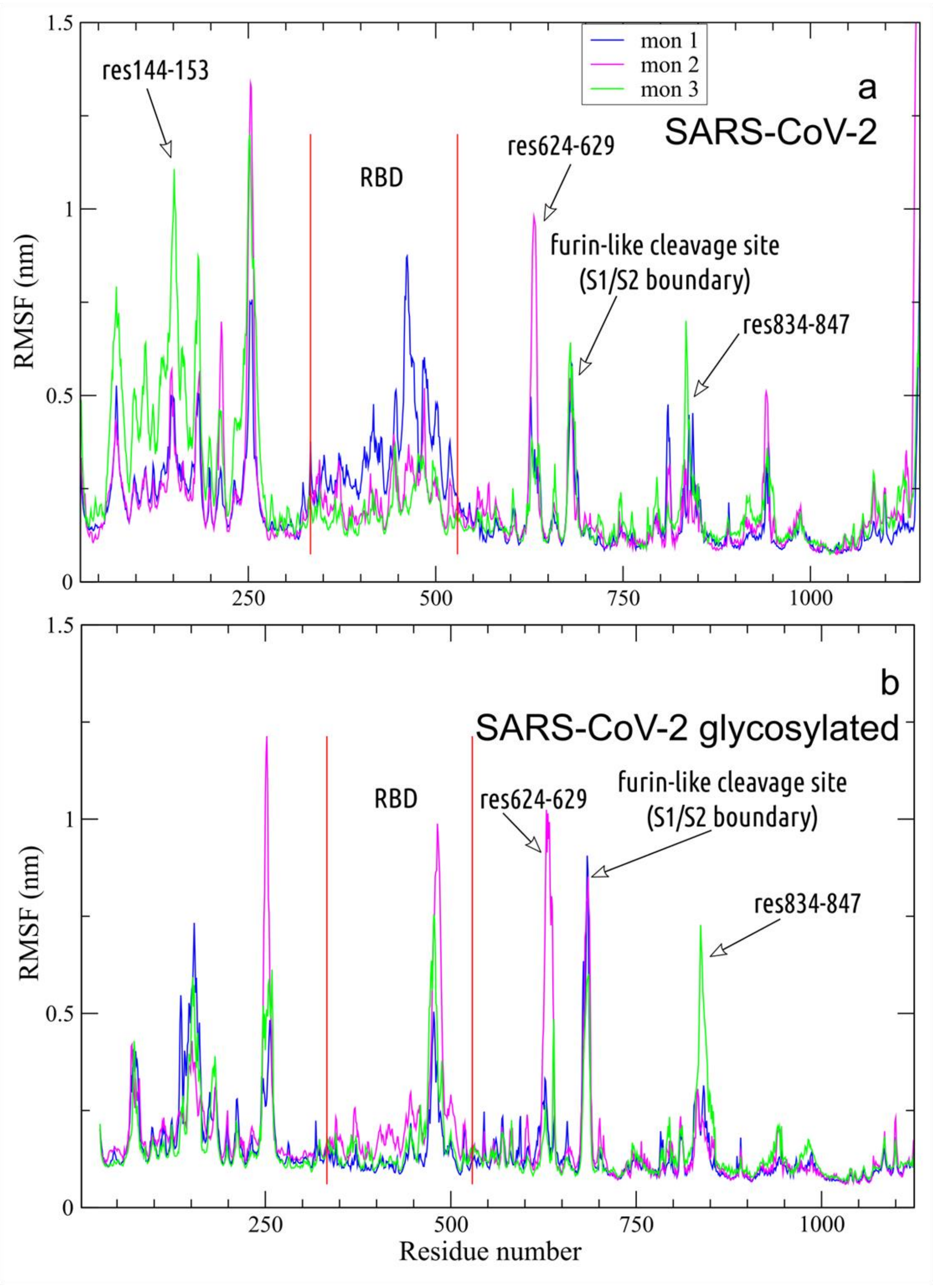

Figure S4. Per-residue RMSF of SARS-CoV-2 in unglycosylated and glycosylated forms are shown in panel a and b, respectively. The three S monomers are colored in blue, magenta and green, respectively. SARS-CoV-2 peak of fluctuations in CTD3 (res624-629), at the S1/S2 cleavage (where SARS-CoV-2 has the newly acquired furin-like cleavage site) and in S2 (res834-847) are conserved in the glycosylated form.

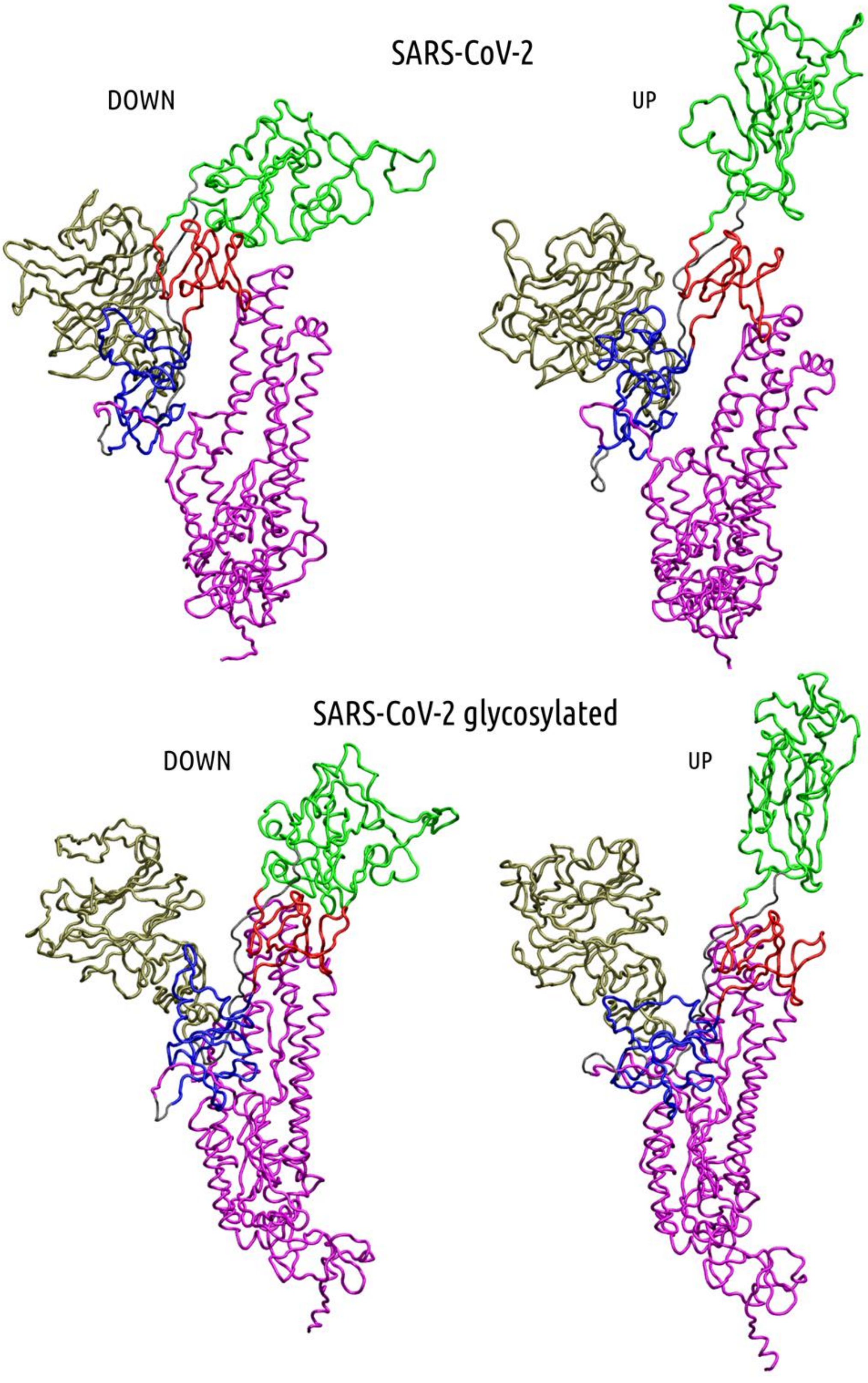

Figure S5. Projection of the S protein MD trajectory along Essential Dynamics (ED) eigenvector 1 for the SARS-CoV-2 system, unglycosylated and glycosylated forms. The two extreme conformations correspond to the up and down RBD conformations (conserved in the glycosylated form) and are indicated as DOWN and UP in the left and right panels, respectively.
