# Supplementary material for: Multiple Recombination Events and Strong Purifying Selection at the Origin of SARS-CoV-2 Spike Glycoprotein Increased Correlated Dynamic Movements
Source: Int J Mol Sci. 2020 Dec 23;22(1):80. doi: 10.3390/ijms22010080 (PMC7794730; doi:10.3390/ijms22010080)
Supplement: Supplementary file 1 [file ijms-22-00080-s001.zip › Supplementary.Movies.zip!/LegendMovies.pdf]

Supplementary movies. RBD rotation in the opening/closing mechanism, functional to receptor recognition, is the most correlated motion in S glycoproteins among all lineages and therefore is the dominant movement along eigenvector 1 (see **movies 1**, **2** and **3** for SARS-CoV-2, Bat-CoV-RaTG13 and Pangolin-CoV-2017, respectively). Domain colors as in Figure 2c-f.

A correlated motion between RBD and residues 834-847, adjacent to the fusion peptide (residues 816-833) is evident in all lineages (see **movies 4**, **5** and **6** for SARS-CoV-2, Bat-CoV-RaTG13 and Pangolin-CoV-2017, respectively). Residues 834-847 and the fusion peptide region are highlighted in cyan and black colors, respectively.

Movie 7. Long-range correlated motions along eigenvector 2, peculiar of SARS-CoV-2, involve residues 624-629, the newly acquired furin-like cleavage site region (residues 681-684), and residues 834-847, adjacent to the fusion peptide (residues 816-833). The three regions are highlighted in cyan color.
